# Sevoflurane Exposure in the Developing Brain Induces Hyperactivity, Anxiety–Free, and Enhancement of Memory Consolidation in Mice

**DOI:** 10.3389/fnagi.2022.934230

**Published:** 2022-06-29

**Authors:** Rui Li, Bei Wang, Xiaohong Cao, Chao Li, Yuhan Hu, Dandan Yan, Yanchang Yang, Liqing Wang, Lingzhong Meng, Zhiyong Hu

**Affiliations:** ^1^Department of Anesthesiology, The First Affiliated Hospital, Zhejiang University School of Medicine, Hangzhou, China; ^2^Department of Anesthesiology, The Children Hospital, Zhejiang University School of Medicine, Hangzhou, China; ^3^Department of Anesthesiology, Jiaxing Hospital of Traditional China Medicine, Jiaxing, China; ^4^Department of Anesthesiology, Lishui Municipal Central Hospital, Lishui, China; ^5^Cell Biology Department, Yale University, New Haven, CT, United States

**Keywords:** pediatric anesthesia, neurotoxicity, sevoflurane, hippocampus, cognition, synapse plasticity

## Abstract

**Background:**

Sevoflurane exposure at brain developmental stages has been reported to induce neurotoxicity and, subsequently, results in learning deficits at the juvenile age. In this study, we aimed to investigate the effects of prior early-age sevoflurane exposure on locomotor activity, anxiety, CA1-dependent learning, and spatial memory, as well as synapse changes in mice.

**Methods:**

Totally, 3% sevoflurane was given to neonatal mice at postnatal day 7 for 4 h. These sevoflurane-treated mice were later subjected to open field and Morris water maze tests at their adult age (postnatal days 60–90) to assess their motor activity and spatial learning ability, respectively. The brain slices of sevoflurane-treated and control mice were examined for dendritic spine density and long-term potentiation (LTP) features following behavior tests (postnatal day 60). Protein levels of N-methyl-D-aspartate (NMDA) receptor subtypes and PSD95 in brain lysate were measured by using immunoblotting at the same age (postnatal day 60).

**Results:**

Prior early-age sevoflurane exposure increased the overall moving distance, prolonged the central-area lingering time, and increased the central-area entries of adult mice. Sevoflurane-treated mice spent more time in the target quadrant during the probe test. An increase of the spine density of pyramidal neurons in the CA1 region was observed in sevoflurane-treated mice. NMDA receptor GluN2A subunit, but not the GluN2B or PSD95, was increased in the brain lysate of sevoflurane-treated mice compared with that of control mice. LTP in the hippocampus did not significantly differ between sevoflurane-treated and control mice.

**Conclusion:**

Exposure to sevoflurane for mice during an early brain developmental stage (P7) induces later-on hyperactivity, anxiety-free, and enhancement of memory retention. These observations shed light on future investigations on the underlying mechanisms of sevoflurane’s effect on neuronal development.

## Introduction

The emergence and development of modern anesthesiology have enabled tens of thousands of patients to tolerate some diagnostic treatments and surgical procedures, which is undoubtedly one of the great achievements in the history of human medicine. Although the sedation of the brain induced by anesthetics was thought to be reversible in the early days, some patients showed delirium, memory loss, and a long period of cognitive decline after anesthesia exposure. The critical period of human neurodevelopment is late pregnancy and infancy, and for rodents, it is within 2 days before birth and within 2 weeks after birth. Therefore, the pharmacological interference of anesthetics is particularly significant and persistent at this stage. In summary, related researchers quickly realized that anesthetic drugs may have a related effect on the neurodevelopment of the immature brain.

Many clinical retrospective cohort studies have shown that the exposure to anesthetics in childhood increases the risk of neurodevelopmental abnormalities, such as learning, cognitive, and behavioral abnormalities, through academic performance or some intelligence and behavioral development scale assessments (Hu et al., 2017; Zaccariello et al., 2019; Ing et al., 2020). Inconsistently, some studies suggested that exposure to anesthesia in early childhood had no significant effect on neurodevelopment. Clinical research cannot rule out the effects of many confounding factors, such as the instability of perioperative vital signs, the variation of anesthetic drugs, and technology, postoperative care, intellectual development education, and so on (Hu et al., 2017; Ing et al., 2020). The establishment of anesthesia animal models allowed researchers to better focus on anesthesia. Some animal studies have confirmed that anesthetics can cause neuronal apoptosis and cognitive dysfunction in the developing brain; however, other reports suggested that anesthetic exposure could even promote brain development (Chen et al., 2015; Hansen, 2015; Sun et al., 2016). These discrepancies may likely due to the differences in the experimental settings (Xu et al., 2016; Chen et al., 2018; Ing et al., 2020), such as the dosage of anesthetic drugs, the exposure time, and the combination.

Animal models have also facilitated the research on effects of anesthetics. The development of the brain involves many complex and time-specific and persistent cellular processes, including the neurogenesis, neuronal differentiation, and neuronal migration. There are many types of anesthetic drugs commonly used in clinical practice, including barbiturates, benzodiazepines, ketamine, and inhaled drugs, such as fluoroether and nitric oxide. The chemical composition of these drugs was diversified, but their neural mechanism of reversible depression was nearly identical: inhibiting neurotransmitter transmission by the N-methyl-D-aspartate (NMDA) receptors or activating neurotransmitter transmission by the gamma-aminobutyric acid (GABA) receptors (Buggy et al., 2000). The formation and stability of synapses are the key factors to ensure the transmission of electrochemical neurotransmitters. Both NMDA and GABA receptors are highly expressed in the hippocampus, which is essential for learning and memory formation. Activation of NMDA receptors is necessary for the formation of long-term memory, whereas activation of GABA receptors inhibits memory formation (Korotkova et al., 2010; Roncacé et al., 2017). Another key feature of brain development is that neurons form functional neural networks through synaptic refinement. Therefore, the interference caused by anesthetic drugs on the occurrence of synapses and the formation of neural circuits may play an important role in developmental disorders. NMDA and GABA receptor signaling pathways have been shown to regulate neuronal proliferation, differentiation, and the formation of neural circuits.

As an inhibitor of NMDA receptors, sevoflurane was the most commonly used inhaled anesthetics for induction and maintenance of pediatric anesthesia due to its quick onset and less airway irritation. More evidence is needed to understand whether and how sevoflurane acts on the central nervous system and the underlying molecular mechanism for neuronal development. To simulate the long-term effects of clinical exposure to anesthesia in infants and young children, herein, we used postnatal 7-day mice exposed to sevoflurane at clinically relevant doses and times. Then, we reared these mice to adulthood and observed behavioral changes by the open field test and Morris water maze; the former observes the motor activity and emotion of mice and the latter measures the spatial memory ability. Based on behavioral results, we further explored the NMDA receptor pathway interrelated long-term effects of sevoflurane on hippocampal functioning, including components changes of NMDA receptor subunits, morphological changes of the synapse, and electrophysiological changes.

## Methods

### Animals

All procedures were carried out in accordance with the National Institutes of Health Guidelines for the Care and Use of Laboratory Animals and were approved by the Animal Advisory Committee at Zhejiang University, Hangzhou, China (Laboratory Animal Management Regulations (2017 Revision)]. The experimental procedure has been reported previously (Shen et al., 2016). Wild-type C57BL/6 mice were purchased from the Jackson Laboratory (Bar Harbor, ME, United States). All mice were housed at the Animal Facility of Zhejiang University where constant temperature and humidity, standard light intensity, and dark/bright rhythm were maintained. A total of 9 L of 46 mice participated in the experiment without accidental casualties.

### Exposure to Sevoflurane

Postnatal 7-day mice were randomly and equally divided into anesthesia group and control group. The anesthesia group was placed in a transparent plastic chamber (length, 20 cm; width, 10 cm; and height, 10 cm) that was wrapped around by the electric blanket to prevent hypothermia during anesthesia. The chamber was continuously flushed with a carrier gas mixture of oxygen and nitrogen (FIO_2_ = 0.6) at 2 L/min. This relative high concentration of oxygen was given during gas exposure to allow spontaneous breath and to avoid hypoxemia (Satomoto et al., 2009). The mice were exposed to 3% sevoflurane for 4 h (Fang et al., 2012). For the control group, one mouse at a time was placed in a plastic chamber flushed with the same carrier gas without sevoflurane for the same period of time. At the end of the gas exposure, the mice from both groups were allowed to grow up to 2 months. A total of 11 men from each group were allocated for the following behavioral experiments due to their constant hormone levels. The other 24 men and women from both cohorts were sacrificed under termination anesthesia, and their brains were collected for the *ex vivo* analysis. The mice that were subjected for brain tissue collection were anesthetized with a small amount of ether before being decapitated to avoid pain, while the mice that performed behavioral experiments were euthanized by excessive inhalation of ether.

### Behavioral Testing

#### Open Field

The open field (45 cm × 45 cm × 45 cm) was made of opaque plastic material. Each mouse was placed individually into the center of the open field and allowed to explore the arena for 10 min. The entry times into the central area, time spent in the central portion (25% of total area), and locomotor activity of the whole field or central area were determined for each mouse. Time spent in the center area was used as an index of thigmotaxis, a measurement often being used for evaluating general anxiety in rodents.

#### Morris Water Maze

One week after the open field test, the mice were subjected to the Morris water maze test. This test was conducted in a closed, quiet, light-controlled environment in the Animal Experiment Center, Zhejiang University. On the first day, each mouse was placed in a 200 cm diameter black plastic pool filled with a depth of 50 cm with opaque water maintained at 23 ± 1°C for acclimatization. On the second day, each mouse was allowed to swim for 3 min to exclude the directionality of the mice and to evaluate the baseline of the number of times they crossed the platform and the time spent in each quadrant. Furthermore, mice were required to locate a 10-cm diameter platform that was submerged 1 cm below the surface of the water by using visual cues around the edges of the pool. In each trial, each mouse was gently put into the pool at one of four randomly assigned positions and allowed to locate the platform. The position of the platform remains the same throughout all training sessions. If the mouse was able to successfully find the platform and stayed on it for 10 s within 60 s, the experiment ended automatically; otherwise, the mouse was gently guided to the platform for 10 s. At the end of each trial, every mouse was gently removed from the water and dried using a paper towel. Each mouse was subjected to four trials per day, each time from one location, and each trial was intermittent for more than half an hour. Then, the formal test was started and lasted for 5 consecutive days (1st–5th test day). This test was videotaped, and the swimming route was tracked and recorded. The averaged latency to reach the platform and swimming path distance of each mouse were calculated daily. Once the water maze test was completed, all mice were allowed to live up to another 30 days for the probe test of water maze test. On the 6th and 30th day (90 days age) of the formal water maze test, the platform was removed, and mice were allowed to swim and the platform crossing times were accounted to assess their long-term memory retention.

#### Golgi Staining

Golgi staining was performed according to the manufacturer’s instructions of the FD Rapid GolgiStain Kit (PK401, FD NeuroTechnologies). In brief, after gas exposure, fresh mice brains were dissected out, immediately immersed in impregnation solution for 2 weeks and then transferred to “Solution C” for 2 days. The 200-μm thick frozen sections were continuously cut off using a microtome (CM30503, Leica, Solms, GER). The sections were then mounted on glass slides coated with 3% gelatin and dried for 2 weeks. The sections were stained with silver nitrate solution “Solutions D and E” and then dehydrated by a series of decreasing concentrations of alcohol. They were finally installed by Permount. Olympus BX53 microscope acquired the images, and the NIH ImageJ software analyzed the neuronal morphology. The investigator was blinded to the control or experimental groups. At least five pyramidal neurons in the hippocampal CA1 region were randomly selected from each mouse. The neurons were then reconstructed by using a camera lucida and a 40 × objective lens (numerical aperture 0.95). The density of apical dendritic spines of a distinct branch was counted by using a 100 × oil-immersion objective lens (NA 1.3). The mean number of spines per micrometer was calculated using the spines from the both sides of dendritic segments.

#### Western Blotting

After gas exposure, pups were allowed to grow up to 2 months, and their hippocampi were obtained and homogenized using a chilled Vibrahomogenizer (Vibra cell, SONICS) containing 2 ml of radio immunoprecipitation assay (RIPA) buffer [1% Triton X-100, 0.1% SDS, 150 mM NaCl, 2 mM ethylene diamine tetraacetic acid (EDTA), 50 mM NaF, 10 mM sodium pyrophosphate, 1.0 mM Na_3_VO_4_, and 1.0 mM Phenylmethylsulfonyl fluoride (PMSF)]. The lysate was then centrifuged at 12,000 *g* for 20 min at 4°C. The supernatant was collected for protein concentration measurement [Pierce™ BCA Protein Assay Kit (Thermo Fisher Scientific, MA, United States)] and was then used for the Western blot analysis. After electrophoresis with 10% polyacrylamide gel electrophoresis (SDS-PAGE), proteins were transferred to polyvinylidene fluoride (PVDF) membranes (Millipore, MA, United States) using a constant voltage of 300 mV for 90 min. The membranes were then blocked in 5% milk in tris buffered saline, Tween (TBST) (25 mM Tris-HCl, 150 mM NaCl, and 0.1% Tween 20, pH 7.4) on a rocker for half an hour at room temperature and then incubated in the primary antibody solution on a rocker overnight at 4°C. Primary antibodies were diluted in blocking buffer solution (5% milk in TBST) with mouse anti-PSD95 (1:1,000; Millipore MAB1596), rabbit anti-GluN2A (1:1,000; Abcam ab133265), or mouse anti-GluN2B (1:1,000; a kindly gift from Jianhong Luo laboratory, the Department of Neurobiology, Zhejiang University). Subsequently, the membranes were washed in TBST for three times before being incubated with horseradish peroxidase (HRP)-conjugated antimouse secondary antibody (1:20,000 dilution in 5% milk in TBST; Thermo Fisher Scientific 31430) and HRP-conjugated antirabbit secondary antibody (1:20,000; Thermo Fisher Scientific 31460) on a rocker for 1 h at room temperature. After being rinsed again with TBST three times, protein bands were visualized using ECL Western blotting detection agentia (Thermo Fisher Scientific). The loading control was detected with mouse anti-β-actin (1:10,000; Sigma-Aldrich A5441) followed by corresponding antimouse secondary antibody. The intensity of the protein bands was quantified with the NIH ImageJ software, and the results were normalized to the corresponding β-actin bands.

#### Slice Recording

Two months after gas exposure, mice (2 months old) were anesthetized with terminated anesthesia of diethyl ether, and their brains were then removed after decapitation. The transverse brain slices (300 μm) containing the hippocampus were obtained rapidly by a tissue slicer (VT 1200S, Leica, GRE); the operation was carried out in oxygenated (95% O_2_/5% CO_2_) and ice-cold modified artificial cerebrospinal fluid (mACSF; in mM: 234 sucrose, 2.5 KCl, 1.25 NaH_2_PO_4_, 0.5 CaCl_2_, 10 MgCl_2_, 26 NaHCO_3_, 11 D-glucose, pH 7.4, 300 mOsm). Furthermore, the brain slices were transferred to a chamber filled with oxygenated ACSF (in mM: 126 NaCl, 2.5 KCl, 1.25 KH_2_PO_4_, 1 MgCl_2_, 2 CaCl_2_, 26 NaHCO_3_, and 10 D-glucose, pH 7.4, 300 mOsm), allowed to recover for 25 min at 32°C, and subsequently for 1 h at room temperature. During the signal recording process, the preoxygenated ASCF liquid was also continuously perfused on the brain slice to ensure the oxygenation requirement of the neurons. Electrode placement was identified based on the location and morphology of the brain slices under infrared differential interference contrast (IR-DIC) optics. ACSF-filled borosilicate glass (A-M system) pipettes (3–6 MΩ) were attached to the headstage of a Heka EPC 1024 amplifier (Heka Elektronik) and carefully adjusted for fast and slow capacitance and series resistance compensation. In the dendritic region of CA1, fEPSPs were evoked by stimulating the Schaffer collateral-commissural pathway (Sccp) by test stimuli (0.066 Hz, 4–5 V, 20 ms) delivered *via* a bipolar tungsten electrode insulated to the tip (tip diameter 50 mm). The slope of field excitatory postsynaptic potential (fEPSPs) measured between the time at which peak amplitude was observed and 1 ms after the application of the stimulus. Extracellular fEPSPs were monitored for 20 min before the evoking stimuli to confirm the stability of the baseline potential. Each slice received evoking stimuli at a high-frequency stimulus (HFS, 100 Hz, 2 trains of stimulation for 1 s applied 20 s apart). The averaged fEPSP slopes at 0, 20, 40, and 60 min after the delivery of evoking stimuli were normalized against the averaged value of fEPSP slopes for 20 min immediately before the delivery of evoking stimuli, and then they were statistically analyzed. Amplified fEPSPs using a laboratory interface board (ITC-16, Instrutech Corp., NY, United States) and stored to disk on a Power Macintosh G3 computer with the acquisition program Pulse, version 8.5 (Heka electronic GmbH, Lambrecht, GER).

### Statistical Analysis

Data are presented as mean ± standard error of the mean (SEM). ANOVA followed by a *post-hoc* Newman-Keul’s test for comparison was used for data analysis of Morris water maze experiments. Unpaired Student’s *t*-test was used for data analysis of all other experiments. All of the data were appreciate with GraphPad Prism (Graph-Pad Software Inc.) and SPSS 22.0 (Chicago, IL, United States). *P* < 0.05 was considered to be a statistical significance.

## Results

### Early Postnatal Sevoflurane Exposure Increased Motor Activity and Deceased Anxiety of Adult Mice

**FIGURE 1:**
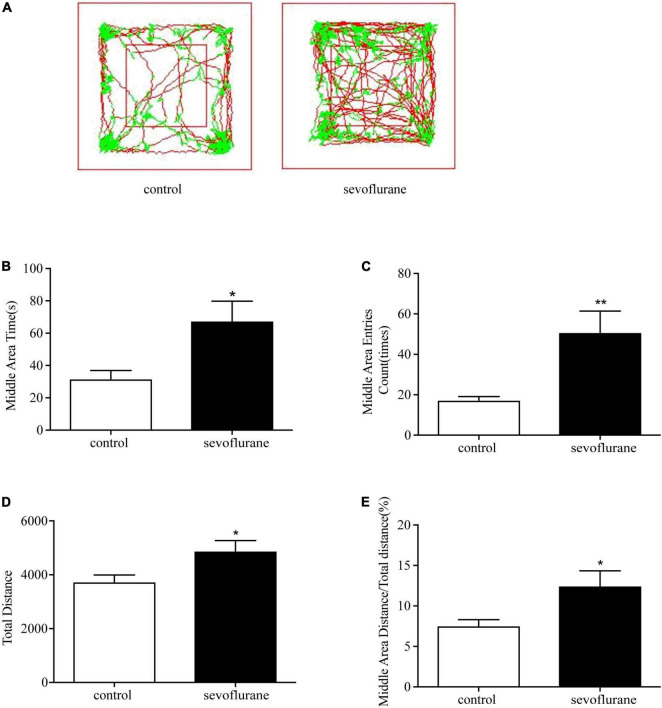
Sevoflurane exposure changed movements in the open field test. Seven-day-old pups were exposed to 3% sevoflurane for 4 h and then allowed to grow up to 2 months. They were then subjected to the open field test. **(A)** The representative movement traces showing the locations of control and sevoflurane mice in the open field test. **(B)** The traveling total distance; **(C)** the crossing times in the central area; **(D)** the time spent in the central area; **(E)** and the traveling distance in the central area. Data are presented as mean ± SEM (*n* = 11). *P < 0.05; **P < 0.01. sevo, sevoflurane.

Open field experiment is one of the most commonly used experimental methods to detect psychological and motor functions. To detect the effect of sevoflurane on the basic motor activity, the open field test was performed to record the moving distance of adult mice during a certain period of time (see section “Methods” for details). We presented a 10-min motor path map of two groups of mice in the openfield (Figure 1A). Results showed that postnatal exposure to sevoflurane increased the overall moving distance (sevoflurane: 4,882 ± 1,031 cm vs. control: 3,736 ± 856 cm; *P* = 0.026; Figure 1B), suggesting that sevoflurane exposure in childhood causes hyperactivity in adulthood. Interestingly, compared with the control group, sevoflurane exposed mice also increased the number of times of entering the central area (sevoflurane: 50.8 ± 35.1 vs. control: 17.3 ± 6.0; *P* = 0.01; Figure 1C), and spent more time in the central area (sevoflurane: 67.6 ± 38.7 s vs. control: 31.7 ± 16.7 s; *P* = 0.018; Figure 1D). These differences were not merely due to the increased motor activity because the ratio of the locomotion distance in the central area over the total was also increased (sevoflurane: 12.5 ± 6.2 vs. control: 7.5 ± 2.6%; *P* = 0.03; Figure 1E), suggesting that sevoflurane exposure during childhood could reduce anxiety-like emotions in unfamiliar environments in mice after adulthood.

### Sevoflurane-Enhanced Consolidate Space Memory in the Morris Water Maze

**FIGURE 2:**
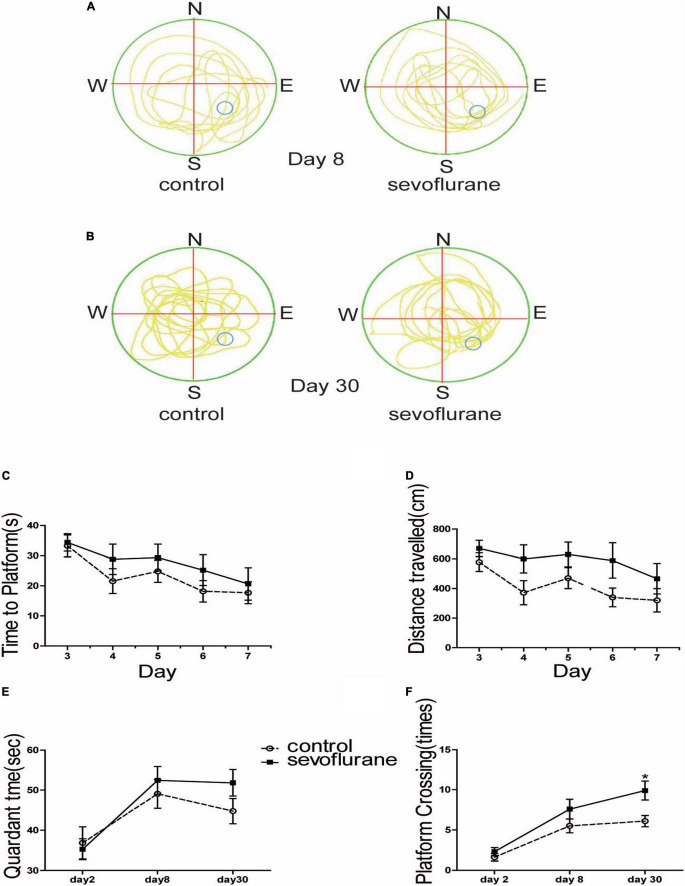
Sevoflurane exposure changed spatial memory in the water maze test. Seven-day-old pups were exposed to 3% sevoflurane for 4 h and then allowed to grow up to 2 months. They were then subjected to the water maze test. **(A)** The trajectory path of day 8; **(B)** the trajectory path of day 30; **(C)** time spent to locate the platform during the 5-day course of the test after the 1st day for acclimatization and 2-day training; **(D)** the distance of swimming path; **(E)** the time spent in the platform region; and **(F)** the platform crossing times. Data are presented as mean ± SEM (*n* = 10). *P < 0.05.

To investigate the effect of sevoflurane on cognition, the Morris water maze test was performed to assess spatial learning and memory. We showed the map of the swimming path of the mouse in the maze just after training (Figure 2A), and the path map of the re-entry into the water maze after a one-month rest period (Figure 2B). We found no statistically significant difference between sevoflurane-treated mice and the control group at 2 months old (*P* > 0.05; Figure 2C). Both swimming distance (*P* > 0.05; Figure 2D) and the time spent in the platform quadrant (*P* > 0.05; Figure 2E) were similar between the treated and control groups. However, the probe test showed significant difference between the anesthesia and control group on the 30th day (sevoflurane: 9.9 ± 3.7 vs. control: 6.1 ± 2.2; *P* = 0.014; Figure 2F) but not on the 6th day (*P* > 0.05; Figure 2E), suggesting that sevoflurane not only interfered with the learning ability of the spatial memory but also enhanced the ability of the mice to consolidate the acquired spatial memory.

### Sevoflurane Increased the Dendritic Spine Density of Pyramidal Neurons

**FIGURE 3:**
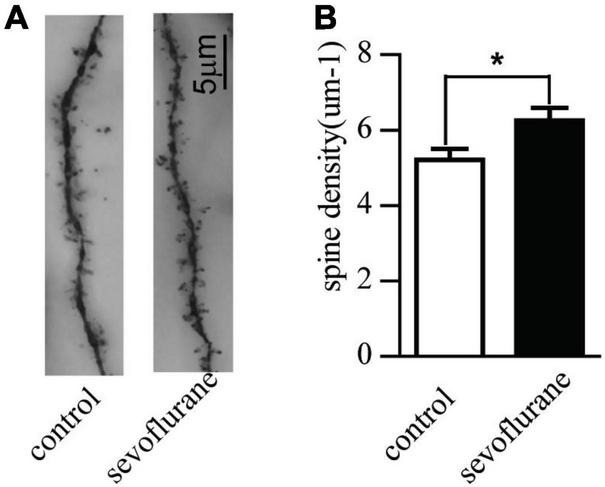
Sevoflurane promoted the development of the pyramidal neurons in the hippocampal CA1 region with Golgi staining. Seven-day-old pups were exposed to 3% sevoflurane for 4 h and then allowed to grow up to 2 months. They were then subjected to the open field and water maze test. After which, their brain was removed for Golgi staining. **(A)** An example of Golgi staining dendritic spine morphology of the tertiary neurons from a Sham-treated or sevoflurane exposed mice; **(B)** the quantitative data of Golgi staining of the dendritic spine density of tertiary neurons. Data are presented as mean ± SEM (*n* = 20 neurons from 3 mice); *P < 0.05.

The formation and stability of synapses are the key factors to ensure the transmission of electrochemical neurotransmitters. To study long-term effects of sevoflurane on neuronal development, we observed synaptic morphology of hippocampal cognitive-related neurons. We counted the numbers of dendritic spines of the pyramidal neurons in the hippocampal CA1 region of 2-month-old mice. Contrary to the control group, early postnatal sevoflurane exposure induced an increase of the spine density of pyramidal neurons (sevoflurane: 6.3 ± 1.8 vs. control: 5.2 ± 1.5; *P* = 0.028) (Figures 3A,B), suggesting sevoflurane promoted the formation of dendritic spines.

### Sevoflurane Increases the N-Methyl-D-Aspartate Receptor GluN2A Subunit in the Hippocampus

**FIGURE 4:**
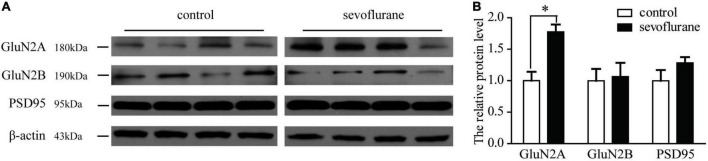
**(A)** Western blotting of GluN2A, GluN2B, and PSD95 in mice of following group: control, sevoflurane. **(B)** Quantification of GluN2A, GluN2B, and PSD95 in mice of the following groups: control and sevoflurane-treated. Sevoflurane increased the GluN2A expression. Seven-day-old pups were exposed to 3% sevoflurane for 4 h and then allowed to grow up to 2 months. They were then subjected to the open field and water maze test. Then, their hippocampus was immediately removed for Western blot analysis. GluN2A expression was significantly increased by sevoflurane exposure. Data are presented as mean ± SEM (*n* = 4); *P < 0.05.

During the process of brain development and maturation, there is a conversion of NMDA receptors from GluN2B to GluN2A. The higher the ratio of GluN2A/GluN2B, the higher the maturity of neurons. To explore whether sevoflurane interfere with this conversion process, the hippocampus from 2-month-old mice was harvested and subjected to Western blot analysis to compare the changes in protein content. Immunoblotting of GluN2A, GluN2B, or PSD95 showed that there was an increase of the levels of GluN2A subunit of NMDA receptor in the hippocampus of the mice after the sevoflurane exposure comparing with the control mice (152 vs. 100%, *P* = 0.01). There was no significant difference in the levels of GluN2B or PSD95 between the sevoflurane-treated mice and the control mice (Figure 4). This indicates that sevoflurane either promotes the above-mentioned conversion process or promotes the expression of GluN2A.

### Sevoflurane Had No Effect on Long-Term Potentiation

**FIGURE 5:**
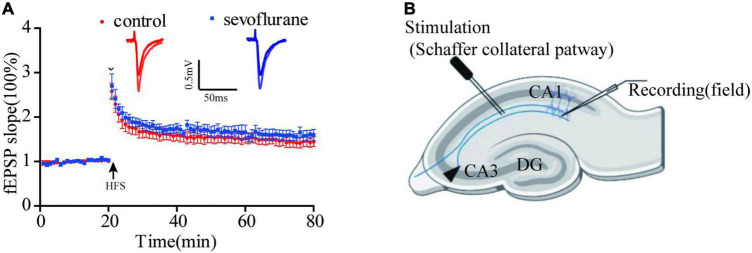
Sevoflurane did not significantly change long-term potentiation (LTP). **(A)** We tested 0, 20, 40, and 60 min after high-frequency stimulation, and the response of sevoflurane exposure group to HFS has a mild increase than that of control group, but it was not statistically significant. **(B)** The position of stimulating and recording electrode in CA1 region. Data are presented as mean ± SEM (*n* = 6 slices from 5 mice for control group and *n* = 5 slices from 5 mice for sevoflurane group).

Long-term potentiation (LTP) of synaptic transmission has been considered as one of the neural foundations of learning and memory, a functional indicator of synaptic plasticity. To evaluate the possibility that sevoflurane exposure may affect neuronal excitability, evoked potentials and synapse plasticity were studied in the hippocampal slices of anesthetized and control group in 2 months after gas exposure. In the control group, 0, 20, 40, and 60 min after HFS delivery, field excitatory postsynaptic potential (fEPSP) were no significant difference between two groups (*P* > 0.05; Figure 5). It seems that sevoflurane does not affect synaptic plasticity.

## Discussion

In our study, we found that 3% sevoflurane exposure for 4 h increased motor activity and promoted anxiety-free and consolidated space memory. Accordingly, compared with the control group, dendritic spine density of pyramidal neurons and the expression of GluN2A subunit were increased in the hippocampus of the sevoflurane group. However, we failed to identify any changes in LTP between the control and sevoflurane-treated group.

In line with our findings, previous studies also showed that neonates exposed to sevoflurane resulted in ADHD-like hyperactivity behaviors (Hu et al., 2017; Liang et al., 2017; Andropoulos, 2018). Although the underlying mechanisms behind these changes remain elusive, it has been reported that general anesthetics can induce dopaminergic receptors (DRs) subtype imbalance, glutamatergic receptors of N-methyl-D-aspartic acid receptor (NMDAR) subtype changes, and brain-derived neurotrophic factor (BDNF) reduction. All these changes, in turn, ultimately resulted in neurobehavioral changes due to various mechanisms including affecting neuronal development, interfering neurotransmitter transmission (Xu et al., 2019), and interrupting connections between the neurons. However, clinically, the GAS study showed no evidence for an increased risk of ADHD after anesthesia (McCann et al., 2019), but other studies indicated that there is an association of anesthetic exposure within the first few years of life and the development of ADHD (Chen et al., 2018). One can argue that neurotoxicity of general anesthetics can occur immediately after exposure and neurological dysfunction can be developed in a few years later. In our previous study, we found that repeated sevoflurane exposure at neonates can lead to impulsive behavior in adulthood (Xie et al., 2020). Moreover, research shows that general anesthesia can lead to hyperactivity behaviors (Xie et al., 2021). It is true that the synaptogenesis in 7-day-old rats is extremely sensitive to external factors, and the toxic effects from anesthetic agents can cause brain functional abnormalities that persist into adulthood. Clinically, ADHD normally appears in old-age children although the pathogenic damage to their brain happens in their early life *per se* (McCann et al., 2019).

Interestingly, the hyperactive and anxiety-like behaviors shown in the open field were not shown in the mirrors water maze experiment. This may be due to the fact that the open field anxiety-like behavior triggered by two main factors, namely, deviated from the original social groups and agoraphobia. In other words, the subject’s response to different experiments is different and will affect the specific experimental results. Besides, previous studies also show that hippocampal damage enhanced spontaneous exercise activity (Yu et al., 2018). But damage to different regions within the hippocampus, such as the ventral and dorsal sides, can also have different effects. There is even an explanation that the anxiety behavior of the ventral hippocampus damage is thought to exist only at the more moderate pressure. Moreover, sometimes, the local area of the hippocampus damage only affects the anxiety-like emotions but not spatial memory capacity (Ju et al., 2019). Therefore, sevoflurane causes many types of behavioral changes, which may be caused by different brain regions and even different substructures. Perhaps, we need to promote mechanisms investigation by separating the above behaviors into related sub-brain regions. Previous studies found that anesthetic exposure in late postnatal life influenced dendritic spine formation and development in mice (De Roo et al., 2009). Indeed, volatile anesthetics of isoflurane, sevoflurane, or desflurane were reported to increase dendritic spine density in the rat medial prefrontal cortex (Briner et al., 2010). This was the case in this study showing that sevoflurane exposure increased dendritic spine density of pyramidal neurons in the hippocampal brain regions. Since excitatory synapses mostly form on dendritic spines (Ju et al., 2019), this may logically lead to change the excitatory synaptic transmission. This may explain our findings that sevoflurane treated mice have higher motor activities and better memory retention. Animal studies had indicated the developmental neurotoxicity of volatile anesthetics, but this study showed that sevoflurane exposure reduces anxiety and enhances memory consolidation in mice, which are accordant with others (Chen et al., 2015). The difference may be mainly due to the different dosage and duration of anesthesia in the established animal models.

In this study, we found that sevoflurane increased the expression of GluN2A but not GluN2B subunit in the whole hippocampus in the adult age after exposure (Figure 4). However, the exposure did not induce any changes of PSD95 and LTP in the CA1 region of the hippocampus. Previous experimental results showed that anesthetics regulate cognitive function mainly by changing the GluN2B content (Dumas, 2005), while the increase in GluN2A content is considered to be a manifestation of long-term memory deficits (Kopp et al., 2007). Moreover, pharmacological evidence indicated that GluN2A are essential mediators of many forms of activity-dependent synaptic plasticity that are thought to underlie higher cognitive functions, such as learning and memory (Paoletti et al., 2013). Increasing the expression of GluN2A did not affect the LTP induced by 100 Hz in CA1 region. Instead, it only affected the LTD induced by fixed range of frequency (Lu et al., 2016). Also, there are still articles notion that the first hour and expression of LTP is still indeterminacy (Nicoll, 2017). Previous studies have shown that in anesthetized mice, neither cognitive performance nor long-term potentiation was impaired 24 h after anesthesia (Haseneder et al., 2013). This is consistent with our electrophysiology. We must point out that this test only detected the CA1 region of the LTP only used a stimulus protocol. Thus, overexpression of GluN2A may be associated with LTP with other induction patterns or other brain regions of long-term memory. Interestingly, sevoflurane anesthesia induced even an improvement of cognitive performance and an elevation of the expression levels of NMDA receptor type 1 and 2B subunits in the hippocampus (Haseneder et al., 2013). In addition, is there a correlation between the increase in GluN2A content and the improvement of memory consolidation ability? These conjectures need to examine in future experiments. This may reflect a regional specific pattern changes but may also indicate that early-life anesthetic exposure can induce differentiating changes in the brain. All these need to be verified in the future study.

This study has several limitations. First, both experimental and control mice the mice pups were maintained under spontaneous ventilation during sevoflurane anesthesia. Sevoflurane suppresses cardiorespiratory function, and, therefore, hypercapnia, hypoxemia, and hypotension likely may occur in this study. Due to technical difficulties, blood gases and blood pressure were not measured and if so, what impact they might have on our current study is unknown. However, it has been reported that previous research demonstrated that there was no significant difference in the rate of hypercapnia during 6 h exposure of sevoflurane at 3% (Goyagi, 2018). Second, only an open field test and the Morris water maze test were used in our study; more behavioral tests should be introduced in our future study. Third, we only selected male mice for behavioral testing, while they do not have the physiological cycle of female mice; we cannot rule out that sex selection may cause gender bias in the behavioral results. Finally, regional specific related molecular mechanisms and their association with behavioral changes are not investigated and warrant further study.

## Conclusion

Totally, 3% sevoflurane exposure for 4 h at the brain development stage increased motor activity and promoted anxiety-free and consolidated space memory in the adult age. Accordingly, the dendritic spine density of pyramidal neurons in the CA1 region and the expression of GluN2A subunit were increased in the hippocampus. However, sevoflurane treatment did not induce any changes in LTP in the hippocampus. Conclusively, this study only describes some of the changes caused by sevoflurane acting on immature brains and raised related questions and directions for subsequent research. The implication of our study may call more studies to further verify the long-term effects of neurotoxicity of general anesthetics.

## Data Availability Statement

The raw data supporting the conclusions of this article will be made available by the authors, without undue reservation.

## Ethics Statement

The animal study was reviewed and approved by the Tab of Animal Experimental Ethical Inspection of the First Affiliated Hospital, Zhejiang University School of Medicine.

## Author Contributions

ZH conceived and designed the study. RL and BW were involved in the study design, experimental operation, data review, data analysis, and writing article. XC and YH analyzed the data and modified the manuscript. CL, DY, YY, and LW contributed for the animal feeding and experimental operation. LM modified the manuscript. All authors read and approved the final manuscript.

## Conflict of Interest

The authors declare that the research was conducted in the absence of any commercial or financial relationships that could be construed as a potential conflict of interest.

## Publisher’s Note

All claims expressed in this article are solely those of the authors and do not necessarily represent those of their affiliated organizations, or those of the publisher, the editors and the reviewers. Any product that may be evaluated in this article, or claim that may be made by its manufacturer, is not guaranteed or endorsed by the publisher.

## Abbreviations

ACSF: artificial cerebrospinal fluid
ADHD: attention deficit hyperactivity disorder
fEPSP: field excitatory postsynaptic potential
HFS: high-frequency stimulation
GABA: gamma aminobutyric acid
LTP: long-term potentiation
mACSF: modified artificial cerebrospinal fluid
NMDA: N-methyl-D-aspartic acid
PSD95: postsynaptic density 95
WT: wild type.
